# Supplementary material for: Development of the petaloid bracts of a paleoherb species, Saururus chinensis
Source: PLoS One. 2021 Sep 2;16(9):e0255679. doi: 10.1371/journal.pone.0255679 (PMC8412408; doi:10.1371/journal.pone.0255679)
Supplement: S2 Table — (DOC) [file pone.0255679.s002.doc]

**S2 Table. Top-hit species distribution**

| **Species** | **Unigene Num** |
| --- | --- |
| Vitis vinifera | 5904 |
| Theobroma cacao | 3948 |
| Oryza sativa Japonica Group | 1547 |
| Cucumis sativus | 1326 |
| Fragaria vesca subsp. vesca | 1154 |
| Glycine max | 974 |
| Solanum lycopersicum | 919 |
| Arabidopsis thaliana | 835 |
| Cicer arietinum | 804 |
| Medicago truncatula | 737 |
