# Supplementary material for: Development of the petaloid bracts of a paleoherb species, Saururus chinensis
Source: PLoS One. 2021 Sep 2;16(9):e0255679. doi: 10.1371/journal.pone.0255679 (PMC8412408; doi:10.1371/journal.pone.0255679)
Supplement: Attachment — Submitted filename: recomendations.docx [file pone.0255679.s007.docx]

The authors compared the morphologies and physiological indices of petaloid and non-petaloid (green) bracts between the perianthless paleoherb S. chinensis in green and white ones. And compared the transcriptome in these two kinds of bracts, identified and verified some differential expressed genes, also discussed the possibility molecular mechanisms of petal development, which is new to us. However, I have some small questions or some mistakes to figure out in this manuscript in below:

1. In Abstract you mentioned the total differential expression genes(DEGs) were 5978, among them up-regulated DEGs were 1770 and down-regulated were 4770, that total among were 5977, not 5978, may this is a simple mistake by careless?
2. All the figures were not having a good quality, they were too blurred, could provide above 300 dpi pictures?
3. You mentioned the B class genes could be interact with each other, what the significance of these genes interaction with each other? And this work has also demonstrated that A- and B-class gene expression are involved in the development of showy bracts to attract pollinators, however, why don’t you detect the A-and B-class genes’ interaction?
4. You should improve your English more fluently for your manuscript.
