# Supplementary material for: Development of the petaloid bracts of a paleoherb species, Saururus chinensis
Source: PLoS One. 2021 Sep 2;16(9):e0255679. doi: 10.1371/journal.pone.0255679 (PMC8412408; doi:10.1371/journal.pone.0255679)
Supplement: Attachment — Submitted filename: coverletter-Response to Reviewers_.doc [file pone.0255679.s008.doc]

"**Development of the petaloid bracts of a paleoherb species, *Saururus chinensis*** "(PONE-S-21-01397).

Dear Editor,

Thank you very much for your comments and suggestions. We have just revised the manuscript according to the comments and suggestions made by yourself and the reviewers. Attached (p2-5) please find a list of corrections and revisions along the lines of the editor and reviewers. For the English, we asked Dr. Zachary Larson-Rabin, University of Wisconsin-Madison to revise the MS for us. Because we want you to see these changes, we have not accepted the track changes he made, believing that you may prefer to see this at your edit. We hope by now that the revised manuscript is acceptable.

The accession numbers were MZ044086-MZ044244, [SRR14085885](https://dataview.ncbi.nlm.nih.gov/object/SRR14085885), [SRR14085884](https://dataview.ncbi.nlm.nih.gov/object/SRR14085884) and [SRR14085883](https://dataview.ncbi.nlm.nih.gov/object/SRR14085883).

Please do not hesitate to get in contact with us as to the revision.

Best wishes,

Sincerely yours,

Yin-He Zhao & De-Zhu Li

Yunnan Agricultural University

Kunming Institute of Botany, Chinese Academy of Sciences

Reviewer(s)' Comments to Author:

Reviewer #1: The authors compared the morphologies and physiological indices of petaloid and non-petaloid (green) bracts between the perianthless paleoherb S. chinensis in green and white ones. And compared the transcriptome in these two kinds of bracts, identified and verified some differential expressed genes, also discussed the possibility molecular mechanisms of petal development, which is new to us. However, I have some small questions or some mistakes to figure out in this manuscript in below:
1. In Abstract you mentioned the total differential expression genes(DEGs) were 5978, among them up-regulated DEGs were 1770 and down-regulated were 4770, that total among were 5977, not 5978, may this is a simple mistake by careless?

We have revised this in the part of Abstract.

2. All the figures were not having a good quality, they were too blurred, could provide above 300 dpi pictures?

Yes, we have revised all the figures.

3. You mentioned the B class genes could be interact with each other, what the significance of these genes interaction with each other? And this work has also demonstrated that A- and B-class gene expression are involved in the development of showy bracts to attract pollinators, however, why don’t you detect the A-and B-class genes’ interaction?

We have revised this in the part of discussion. However, the corresponding ‘ABCDE’ model maintains that class A+E genes specify sepals, A+B+E specify petals (Theißen,2001; Ditta et al.,2004;Theißen et al., 2016). a complex of one AP1 and one SEP protein together with one of each of the class B proteins APETALA3 (AP3) and PISTILLATA (PI) determines petal identity. First, the class B proteins APETALA3 (AP3) and PISTILLATA (PI) could form protein dimerization, therefore, it is very important for the B class genes could be interact with each other, and then could be interact with a complex of one AP1 and one SEP protein dimerization. If the class B proteins APETALA3 (AP3) and PISTILLATA (PI) could not form protein dimerization, it would be senseless to add A-class gene, because there is no the floral quartet model (FQM) form to identify the petal.

4. You should improve your English more fluently for your manuscript.

We have asked Dr. Zachary Larson-Rabin, University of Wisconsin-Madison to revise the MS for us.

Reviewer #2: Zhao et al. studied the development of petaloid bracts in Saururus chinensis. The topic is very interesting, and the results at the anatomy, physiology and gene expression levels support their conclusions. However, several places in result part were confusing, and need be clarified, and some places in Methods were even wrong. Here, I list some:

1.“RNA-seq abundance analysis identified 43,463 genes that were found to be differentially expressed in S. chinensis bracts. Of these, 5,978 showed strong differential expression, of which 1,770 were up-regulated and 4,207 down-regulated in green compared to white bracts”. That means that 90% genes identified in this study were differentially expressed. What is the criteria to design “differentially expressed gene” and “strong differential expression” used in this study?

We have revised this in the part of Abstract.

2. “Clean reads from all samples were pooled together, and were further assembled de novo using the Trinity program [23], resulting in 86,532,094 total reads (10,816,511,750 bps) with an average length of 908bp, an N50-value of 1582 bp, andwith 13,821 unigenes (30.71%) longer than 1000 bp (Table 1). Your sequencing method (single end or pair end and length) need be clarified in Material and Methods, “average length of 908bp” is not the length of clean reads.
   We have revised this in the part of Material and Methods and Results. our sequencing method was pair end and average length of 908bp is unigenes, not the length of clean reads.

3.“22,688 unigenes were assigned to one or more ontologies. 6,727 unigenes were grouped under biological processes, 5,805 unigenes were grouped as cellular components, and 7,445 unigenes were grouped as having a role in molecular functions”. The total unigene number designed to three GO category is less than 22688, even though certain genes can be designed to 2 or 3 GO category.
We have revised this part of the paper.

4.“Comparing the bract tissues’ transcript profiles with the transcript profiles of developing seedling leaves identified 166 bract DEGs”. In the following sentence, authors showed the up/down regulated DEGs of different development stages compared with seedling leaves, what stage did the 166 DEGs specific to?

We have revised in Table S5. Both the green-to-white transition and the white -to- green transition

In Methods part:
5.“According to the Illumina manufacturer’s instructions, poly(A)+ RNA was purified from 5 mg of pooled total RNA from petaloid-bract and green-bract cDNA libraries using oligo(dT) magnetic beads”. 5 mg is misspelled or not? How polyA RNA can be isolated from cDNA libraries?
We have revised this part of the paper.

6.In “Screening of differentially expressed genes” part, “Unigenes that showed different expression levels between the two samples were subjected to GO function analysis and KEGG pathway analysis”, what is the two samples?
We have revised this part of the paper. Two samples are petaloid-bract and green-bract

7.“Following amplification, melting curve analyses were performed at 95°C for 10 min, followed by 40 cycles of 95°C for 30 s, 60°C for 30 s, and 72°C for 20 s”, is this for melting curve analysis?
Yes, this is for melting curve analysis

Other issues:

8.Labelling (A, B….) on figures are not clear.

We have revised on figures.

9.Sequencing data should be deposited into databases for publicly available.
Yes, we have deposited into databases.

10.Authors should pay attention to the grammatical errors, such as “The wild is found mainly in moist sites in southern China from sea level to 1700 m [29]”.

We have revised this part of the paper.
