# Supplementary material for: Development of the petaloid bracts of a paleoherb species, Saururus chinensis
Source: PLoS One. 2021 Sep 2;16(9):e0255679. doi: 10.1371/journal.pone.0255679 (PMC8412408; doi:10.1371/journal.pone.0255679)
Supplement: Attachment — Submitted filename: renamed_3c372.docx [file pone.0255679.s009.docx]

Dear Editor，

I think you can accept this manuscript, I have no other questions.
