# Supplementary material for: Development of the petaloid bracts of a paleoherb species, Saururus chinensis
Source: PLoS One. 2021 Sep 2;16(9):e0255679. doi: 10.1371/journal.pone.0255679 (PMC8412408; doi:10.1371/journal.pone.0255679)
Supplement: Attachment — Submitted filename: Response to Reviewers.doc [file pone.0255679.s010.doc]

# **"Development of the petaloid bracts of a paleoherb species, *Saururus chinensis* "(PONE-D-21-01094R1).**

Dear Editor,

Thank you very much for your comments and suggestions. We have revised the manuscript according to the comments and suggestions made by yourself and the reviewers. Attached (p2-3) please find a list of corrections and revisions along the lines of the editor and reviewers. We hope by now that the revised manuscript is acceptable.

Please do not hesitate to get in contact with us as to the revision.

Best wishes,

Yin-He Zhao & De-Zhu Li

Yunnan Agricultural University

Kunming Institute of Botany, Chinese Academy of Sciences

Reviewer #1: This manuscript has been fully satisified my questions about what I am asking, and this manuscript has some new ideas about its topics, so I advise to accept it for publication on this journal.

Reviewer #2:

1. In abstract and result parts, “RNA-seq abundance analysis identified 43,463 genes that were found to be differentially expressed in S. chinensis bracts. Of these, 5,978 showed strong differential expression, of which 1,770 were up-regulated and 4,207 down-regulated in green compared to white bracts”. You claimed “43,463 genes that were found to be differentially expressed in S. chinensis bracts’, then, you claimed “5,978 showed strong differential expression”, what is the criteria to design “differentially expressed gene” and “strong differential expression” used in this study?

We have revised in Manuscript.

The criteria to design “differentially expressed gene” and “strong differential expression” used in this study was that “differentially expressed gene” was different expression between green and white bracts, and the “strong differential expression” was FDR < 0.001 and an absolute value of the log2 ratio >1 as the threshold to determine the significant difference in gene expression. We thought the significant difference expression was strong differential expression in this study and have revised the strong differential expression to the significant difference expression in abstract and result parts.

2. Both RNA-Seq and microarray were used in this study, differentially expressed genes (DEGs) were screened with each method, how about the consistence between the DEGs of the two method?

The consistence between the DEGs of RNA-Seq and microarray was good.

3. “Scanning Electron Microscopy (SEM) and transmission electron microscope (TEM)” and other subtitles in method are not properly used.

Yes, we have revised in method of Scanning Electron Microscopy (SEM) and transmission electron microscope (TEM) observation and Stomatal conductance (gs) and Chlorophyll concentrations measurement.
